# Secondary oral syphilis presenting as a tumor-like lesion on the lower lip

**DOI:** 10.1590/S1678-9946202466006

**Published:** 2024-02-05

**Authors:** Norberto Sugaya, Dante Migliari

**Affiliations:** 1 Universidade de São Paulo, Faculdade de Odontologia, Departamento de Estomatologia, Clínica de Estomatologia, São Paulo, São Paulo, Brazil

**Keywords:** Syphilis, Atypical oral manifestation, Micropolyadenopathy, Diagnosis

## Abstract

This study presents a case of atypical manifestation of secondary syphilis. Diagnosis was initiated prompted by the patient’s complaint of a lower lip lesion, present for three months, resembling a malignant neoplasm. The lesion, a 3 cm (diameter) ulcerated nodule, arising from conjunctive tissue, raised concern. However, further physical examination revealed additional clinical features, including cervical micropolyadenopathy and erythematous skin lesions, prompting a reevaluation of the diagnosis, most likely secondary syphilis. These findings led to a serological investigation, which, ultimately, confirmed the diagnosis of syphilis. The case underscores the importance of recognizing syphilis as a formidable imitator, posing challenges in establishing differential diagnoses of mucocutaneous diseases.

## INTRODUCTION

Encountering challenging diagnoses, especially in Oral Medicine, is not uncommon, particularly when dealing with manifestations of syphilis. This disease can present a diverse range of clinical appearances and effectively mimic various mucocutaneous conditions^1-4^. This article describes a specific case in which the observed lesion bore a closer resemblance to a malignant neoplasm than an infectious disease. Nevertheless, further clinical examination revealed additional characteristics, such as cervical micropolyadenopathy and skin lesions, ultimately leading to the diagnosis of secondary syphilis.

## CASE REPORT

**Figure 1:**
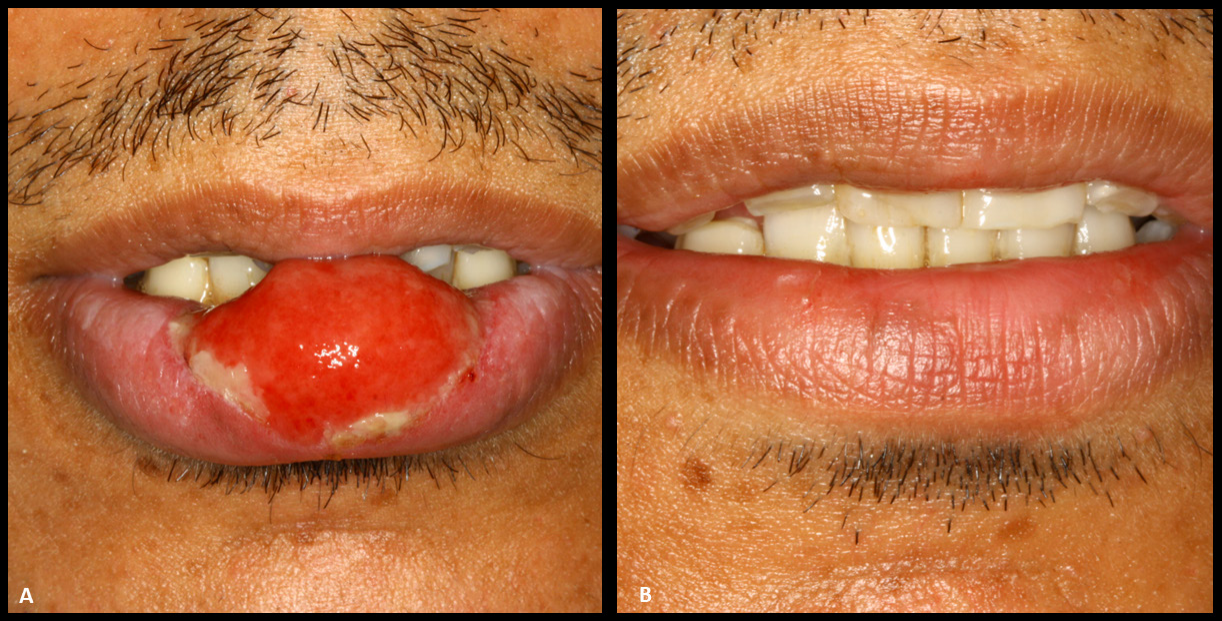
- A) An extensive nodule-ulcerative lesion on the lower lip. Note that the lesion emerges from conjunctive tissue and is covered by a layer of hemorrhagic fibrin; B) The patient’s lip exhibiting a normal aspect three weeks after the second injection of benzathine penicillin.

A 33-year-old male was referred to our clinic with a 3-month history of an ulcerated, nodular-like lesion in the middle of his lower lip. The lesion, approximately 3 cm in diameter, exhibited a rounded shape and a surface covered by a layer of hemorrhagic and purulent fibrin (Figure 1A). Despite its resemblance to a malignant neoplasm, the lesion seemed to arise from conjunctive tissue and caused moderate pain. Palpation revealed no induration of the surrounding tissue, and the nodular area exhibited slight fibrous characteristics. Cervical palpation indicated numerous small lymph nodes in the submental and submandibular chains.

**Figure 2:**
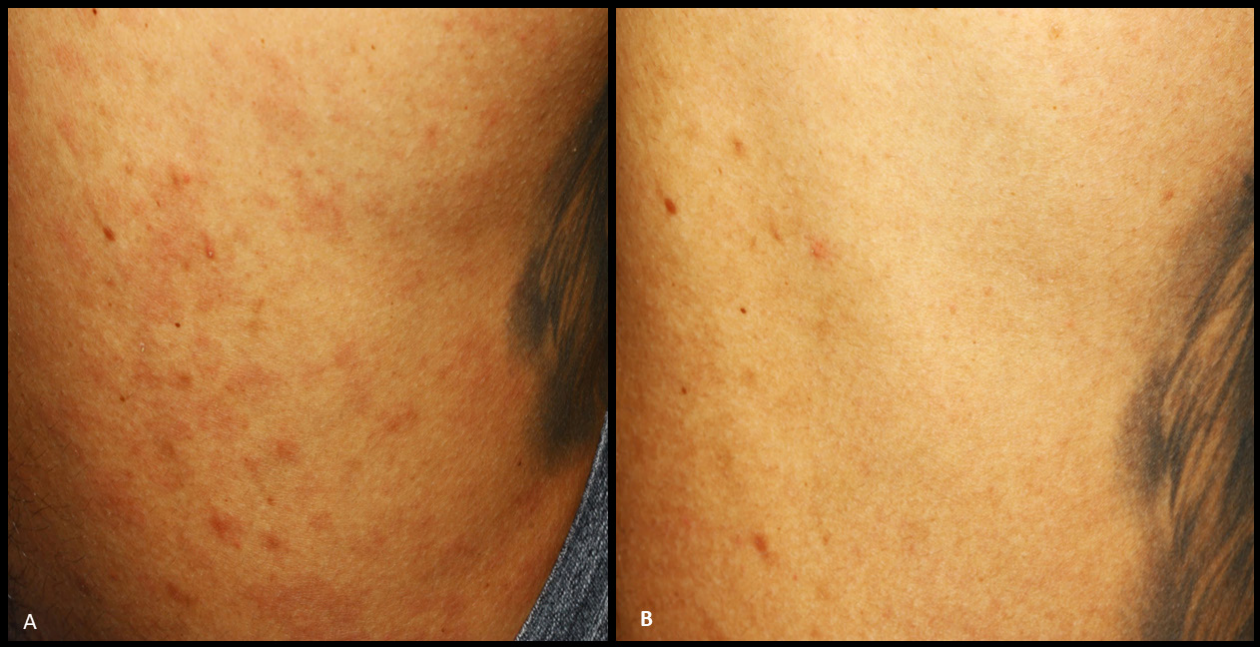
- A) Erythematous papules and plaque lesions covering almost the entire skin surface of the patient’s back; B) A substantial resolution of the lesions following treatment.

Upon further inquiry, the patient disclosed additional clinical manifestations, reporting lesions on his back skin surface. Examination revealed extensive erythematous papules and plaque lesions covering almost the entire back (Figure 2A). The patient denied any genital lesions. Notably, he was a smoker, a moderate alcohol consumer, and regularly used illicit drugs, including crack. He had no other reported medical conditions and was not using systemic or topical medications.

These comprehensive clinical findings raised suspicions of syphilis, particularly owing to the multiple lymphadenopathies and skin lesions, despite the patient’s denial of engaging in sexual behaviors that increased the risk of sexually transmitted diseases. Syphilis serology results confirmed the diagnosis (Venereal Disease Research Laboratory: 1/32; specific antibody anti-*Treponema pallidum*: 20.29), whereas HIV and hepatitis C tests returned negative results. Subsequently, the patient was referred to a sexually transmitted disease center for treatment and contact tracing. The prescribed treatment involved two doses of intramuscular benzathine penicillin G (2.4 million units each), administered two weeks apart. Substantial improvement in the lesions was observed three weeks after the last injection (Figures 1B and 2B), with follow-up scheduled every three months to monitor the decline in titer.

## DISCUSSION

Syphilis, a systemic infection caused by *Treponema pallidum*, poses diagnostic challenges due to its ability to mimic a wide range of mucocutaneous lesions, requiring thorough examination. In this case, the initial clinical impression resembled a neoplastic lesion, most likely a mesenchymal malignant type, given the ulcerated, nodular-like appearance. Despite considering a malignancy diagnosis, cervical palpation revealed numerous small lymph nodes, indicative of micropolyadenopathy and inconsistent with neoplasms. This discovery shifted our diagnostic focus, leading us to consider an alternative, likely infectious, in etiology. Syphilis emerged as a primary contender in the differential diagnosis, especially with the additional finding of extensive erythematous maculopapular skin lesions. A clinical diagnosis of secondary syphilis was considered, later confirmed via serological investigation.

The highlight of this case, characterized by a tumor-like lesion, is the presence of a diffuse cervical micropolyadenopathy. Unlike its primary stage, in which an enlarged satellite lymph node typically accompanies the initial painless, solitary, indurated, ulcerated lesion (chancre), secondary syphilis does not consistently exhibit cervical micropolyadenopathy. Multiple lymphadenopathies can signal various diseases, typically infectious in nature^5-7^. Although the cutaneous lesions in this case played a crucial role in the diagnosis of syphilis, the presence of micropolyadenopathy itself, even in the absence of cutaneous lesions, warrants consideration and investigation for syphilis.

Another possible but very improbable characterization of this case, based on the appearance of the lip lesion, with primary syphilis occurring in combination with its secondary stage, relies on the hypothesis that the patient was reinfected during the secondary stage of the disease. This was almost impossible to verify given that the patient denied engaging in risky sexual behaviors that could have exposed him to a sexually transmitted disease. Moreover, a main barrier to this hypothesis was that the clinical aspect of the lip lesion was far from displaying any similarity to that of the usual chancre lesion, which characterizes the primary form of syphilis manifestation^5,8^.

## References

[1] Reinehr CP, Kalil CL, Reinehr VP (2017). Secondary syphilis: the great imitator can’t be forgotten. Rev Assoc Med Bras.

[2] Sakthivel P, Kakkar A, Sharma SC, Panda S (2018). Mucocutaneous secondary syphilis: ‘the great imitator’. Am J Med.

[3] Smith MH, Vargo RJ, Bilodeau EA, Anderson KM, Trzcinska A, Canterbury CR (2021). Oral manifestations of syphilis: a review of the clinical and histopathologic characteristics of a reemerging entity with report of 19 new cases. Head Neck Pathol.

[4] Lampros A, Seta V, Gerhardt P, Isnard C, Husson C, Dupin N (2021). Oral forms of secondary syphilis: an illustration of the pitfalls set by the great imitator. J Am Acad Dermatol.

[5] 3<sup>rd</sup> Hook EW (2017). Syphilis. Lancet.

[6] Park SY, Kang JH, Roh JH, Huh HJ, Yeo JS, Kim DY (2013). Secondary syphilis presenting as a generalized lymphadenopathy: clinical mimicry of malignant lymphoma. Sex Transm Dis.

[7] Bickford DD, Johnson P, Brahmbhatt N, Kroft S (2023). Cervical syphilitic lymphadenitis in a 29-year-old female: a case report. Cureus.

[8] Thums MA, Koth VS, Figueiredo M, Cherubini K, Salum FG (2021). Oral manifestations of syphilis: an epidemiological study in southern Brazil. Aust Dent J.

